# Sand in the Stomach of Ruminants

**Published:** 1897-07

**Authors:** 


Sand in the Stomach of Ruminants.—In the summer of 1896 the frequent showers and extraordinarily heavy gusts of rain caused a good deal of sand to get into the fodder. When this green fodder was fed to the cattle disturbance in digestion was very frequent in ruminants in this neighborhood. The symptoms are essentially those of traumatic indigestion—loss of appetite, constipation, or even violent diarrhœa, general dulness, difficulty in breathing, and general weakness, which sometimes shows itself in an unsteady gait. An especial symptom was the arching of the backs. The paunch (first stomach) seems very hard to the touch and shows no movement of any kind. I have always given the usual treatment for indigestion, especially tinct. veratri in double doses. I have always had good results, and up to date have only had to kill one cow. In the stomach about six kilogrammes of sand were found, and stones to the size of a pea.—*Thierärztliche Centralblatt*, November 15, 1896.

